# Advances in the Plant Microbiome: Rhizosphere, Endosphere, and Phyllosphere

**DOI:** 10.3390/microorganisms13112581

**Published:** 2025-11-12

**Authors:** Gustavo Santoyo

**Affiliations:** Institute of Chemical and Biological Research, Universidad Michoacana de San Nicolás de Hidalgo, Morelia 58030, Mexico; gustavo.santoyo@umich.mx

## 1. Introduction

The continuous growth of the global human population demands sustainable production systems that move away from synthetic fertilizers, pesticides, and other agrochemicals, which pose serious toxicological, environmental, and public health risks [1]. Consequently, it is essential to refocus attention on microorganisms that can be regarded as an extension of the plant genome. Plants have the remarkable ability to interact and associate with a myriad of microorganisms that enable them to grow, reproduce, and achieve greater fitness under different environmental conditions [2]. Although there are various microbial taxa capable of causing harm to plants, the vast majority play beneficial roles. These beneficial microorganisms associate with different plant tissues, such as the underground part (which includes the root system) and the aerial parts (stems, leaves, and flowers). They can also penetrate internal tissues and produce metabolites (e.g., phytohormones, siderophores, antibiotics) and enzymes (e.g., cellulases, chitinases, phosphatases, and ACC deaminase) that stimulate the growth and fitness of the host. These zones of interaction between microorganisms and their plant host are referred to as the rhizosphere (the soil region influenced by plant roots), endosphere (the internal tissues of the plant and seeds), and phyllosphere (the surface of leaves and aerial parts) [3,4]. Some of these microorganisms have been referred to as plant growth-promoting microorganisms (PGPMs) [5].

For decades, global research has demonstrated that this plant microbiome—defined as the entire community of microorganisms along with their genetic, metabolic, and enzymatic functions—can be harnessed to enhance agricultural productivity by improving plant growth, health, nutrient uptake, stress tolerance, and/or resistance to pathogens [6].

This editorial article introduces the Special Issue “Advances in the Plant Microbiome: Rhizosphere, Endosphere and Phyllosphere”, which comprises 11 publications highlighting the progress and significance of plant microbiome research and its beneficial functions.

## 2. Overview of Published Articles

One of the most relevant topics in recent years has been understanding the factors that regulate and shape plant microbiome communities. In this context, the work by Li et al. (Contribution 1) conducted a metagenomic analysis of the microbiome associated with tomato leaves and fruit pericarp, focusing on the effects of domestication and genetic improvement. The study included more than one hundred germplasms from three tomato clades: *Solanum pimpinellifolium* (PIM), *S. lycopersicum* var. *cerasiforme* (CER), and *S. lycopersicum* (BIG). In leaves, an increase in *Firmicutes* and *Bacteroidetes* was observed, while *Actinobacteria* decreased and *Proteobacteria* remained stable. Within the latter phylum, *Gammaproteobacteria* decreased in abundance, whereas *Alphaproteobacteria* and *Betaproteobacteria* increased. In the fruit pericarp, *Firmicutes* was less abundant in CER and BIG groups than in PIM, whereas *Candidatus Micrarchaeota*, *Actinobacteria*, and *Bacteroidetes* were more abundant. The microbial structure followed patterns similar to those found in leaves, with a decline in *Gammaproteobacteria* and an increase in *Alphaproteobacteria* and *Betaproteobacteria*. However, domestication had a smaller effect on the alpha diversity of the fruit pericarp microbiome than on that of the leaf, where *Methylobacterium*, *Vibrio*, and *Chamaesiphon* increased, and *Plantactinospora*, *Paraburkholderia*, *Ictalurivirus*, decreased during the domestication and breeding processes. Overall, the study suggests that domestication and genetic improvement modulate the structure of the tomato-associated microbiome, although their impact on crop productivity remains to be assessed.

In another study conducted by Zhu and colleagues (Contribution 2), targeted sequencing of fungal ITS regions and bacterial 16S rRNA genes was performed to characterize and compare the dynamics of bacterial and fungal communities in the rice root endosphere and to contrast them with the microbial diversity of the soil in organically and conventionally cultivated paddy fields. The results suggest the occurrence of temporal changes in the alpha diversity of root and soil microbial communities across different paddy systems. Furthermore, agricultural management practices were found to shape the architecture of microbial communities throughout four developmental stages: tillering, elongation, early ripening, and maturing. Principal Coordinate Analysis (PCoA) revealed that both bacterial and fungal communities in the root endosphere and bulk soil clustered distinctly into two groups according to the type of paddy management, highlighting its relevance. In conclusion, this study demonstrates that organic management promotes rice health and sustainability by favoring nitrogen-fixing bacteria such as *Bradyrhizobium* and *Azospirillum* (among other groups), which may enhance nutrient uptake and pathogen resistance, whereas conventional management relies on chemical fertilizers and pesticides, enriching microorganisms capable of degrading synthetic compounds.

In a different work by Wang et al. (Contribution 3) published in this Special Issue, a 10-year continuous experiment was conducted in which a maize–soybean rotation (control group), alfalfa (legume), and wheat (poaceae) were cultivated to assess the impact of different cropping systems on soil microbial communities and their functional attributes. At the same time, the physical and chemical properties of the soil samples were evaluated and correlated with the rhizosphere microbial communities. The results showed that the relative abundance of the order *Cyanobacteriales* was higher in wheat than in alfalfa, and approximately 80% of the bacterial genes in the roots of the three crops were related to metabolism. Similarly, a comparable proportion of fungal genes were associated with saprotrophic groups, suggesting that the nutritional sources for fungi mainly depend on the decomposition of organic matter derived from plant residues and roots.

Additional findings from the study revealed significant correlations between soil fertility and bacterial diversity. For example, peroxidase activity was positively correlated with the Shannon and Simpson diversity indices for bacteria (except for rhizobia such as *Mesorhizobium*), while the presence of *Fusarium* showed a positive correlation with the total potassium content in the soil. Among soil fertility indicators, both total potassium and available phosphorus were positively correlated with peroxidase activity. The authors concluded that further research is needed to better understand how soil microbial communities adapt to agricultural practices and to assess how their expansion affects the balance of the agroecosystem.

Agricultural practices and crop rotation modify the composition and function of the soil microbiome (as previously observed), with rotational systems promoting greater microbial diversity, soil fertility, and plant resilience under adverse conditions. In this regard, studies such as that of Khan and co-authors (Contribution 4) have shown that pathogen diversity and abundance can also be influenced. They evaluated this by analyzing the incidence of Verticillium wilt and the soil microbiome composition in cotton (*Gossypium* spp.) fields from the northern and southern regions of Xinjiang, China. This disease, caused by soil-borne *V. dahliae*, infects vascular tissues and leads to severe crop losses. Interestingly, crop rotation increased the abundance of genera such as *Sphingomonas* and *Pseudogymnoascus* while reducing that of *V. dahliae*. According to the authors, this practice also enhanced the complexity of bacterial interaction networks in the soil, suggesting that crop rotation could serve as an effective strategy to reduce the incidence of Verticillium wilt in cotton.

Some abiotic factors, such as the presence of heavy metals in agricultural soil systems, can modulate microbial communities, particularly those associated with crop plants. Such is the case of cadmium (Cd), as demonstrated by the work of Liu and colleagues (Contribution 5), who found that this contaminant causes significant stress in Giant Duckweed (*Spirodela polyrhiza*) fronds, modulating its microbiome. The phyllosphere microbiome of this aquatic plant was observed to be restructured under Cd concentrations of 0, 1, and 10 μM. Genera such as *Herbaspirillum*, *Enterobacter*, and *Pantoea* increased significantly in the presence of Cd. Functions such as the nitrate/nitrite transporter NarK, signal transduction mechanisms, and ion channel proteins were detected through inferences using PICRUSt2. These identified taxa and functions could serve as markers of contaminated sites or to enhance phytoremediation based on *S. polyrhiza*–microbiome interactions.

In this same context, Khatoon and coauthors (Contribution 6) present a review of microbial contributions to soil phytoremediation, with an emphasis on agricultural soils. Heavy metals such as Cd, lead (Pb), mercury (Hg), and chromium (Cr), as well as metalloids like arsenic (As), resulting from anthropogenic pollution, can be remediated through various phytoremediation processes (including phytoextraction, phytodegradation, phytostabilization, phytovolatilization, rhizofiltration, and rhizodegradation) with the help of the microbiome, which promotes plant growth even under these stressful conditions.

Drought and heat stress are other abiotic factors that can reduce crop yield, where the microbiome can help mitigate negative effects. This was investigated by Magaisa et al. (Contribution 7), who subjected six genotypes of sorghum (*Sorghum bicolor*) plants to these two stresses induced by climate change and evaluated the role of the rhizobacteriome in productivity. The authors found a high diversity of rhizobacteria associated with the sorghum genotypes, and grain yield performance varied according to the analysis of 16S rRNA amplicon sequences. Certain bacterial groups such as *Chloroflexi* (class = *Chloroflexia*) and *Firmicutes* (class = *Bacilli*) were significantly associated with improved grain production. Isolating and identifying these groups could be the next step toward developing bacterial inoculants with high potential as biofertilizers to sustainably increase crop yields. As demonstrated by Msiza et al. (Contribution 8), who selected a group of symbiotic rhizobacteria (rhizobia) from 11 different legume plant species. The results showed that certain plant species, such as cowpea (*Vigna unguiculata*), Bambara groundnut (*Vigna subterranea*), and Kersting’s groundnut (*Macrotyloma geocarpum*), benefited from interactions with nodulating and nitrogen-fixing rhizobia.

Another benefit of the plant microbiome, in addition to directly promoting plant growth through phytohormone production and enhanced nutrient uptake, is the biological control of fungal pathogens. In the study by Jin et al. (Contribution 9), inoculation with *Bacillus velezensis* L11-7 was evaluated as a potential biocontrol agent against stem basal rot of passion fruit (*Passiflora* spp.) caused by *Fusarium solani*. The results demonstrated that strain L11-7 significantly reduced the severity of basal rot in tissues such as the stem by 92% and also exhibited antagonistic activity against other evaluated plant pathogenic fungi. When assessing antifungal mechanisms, L11-7 was found to possess cellulase, glucanase, and protease activities. Genome sequencing revealed genes related to the production of antimicrobial compounds such as fengycin, surfactin, macrolactin H, bacillaene, and difficidin, while other traits associated with plant growth promotion, such as auxin production and nitrogen fixation, remain to be analyzed.

Other groups of microorganisms that play an essential role in promoting plant growth and enhancing host plant nutrition include mycorrhizal fungi. In the study by Liu et al. (Contribution 10), it was found that the mycorrhizal fungus *Humicolopsis cephalosporioides* is important for producing cellulases and providing carbohydrates to the plant *Cheilotheca humilis*. In addition to identifying this potential beneficial role of *H. cephalosporioides*, other bacterial genera, such as *Rhizobium*, *Herbaspirillum*, and members of Cyanobacteriota, were detected in the metagenomic analyses associated with the host plant, showing that they are part of the core microbiome and have potential to form networks of beneficial interactions. Interestingly, the authors also conducted experiments showing that mycorrhizal helper bacteria (MHBs), belonging to the genera *Rhizobium* and *Pseudomonas*, possess mechanisms for phosphate solubilization, iron-chelating siderophore production and nitrogen fixation.

In the same context, Condori et al. (Contribution 11) evaluated the role of inoculating the nitrogen-fixing bacterium *Azospirillum brasilense* on various phytometric parameters of purple maize (*Zea mays* L.) with the aim of reducing and supplementing nitrogen fertilizer inputs in this crop. Inoculation with *A. brasilense* significantly improved growth, biomass, nitrogen content, and yield in maize. Notably, inoculation combined with 90 kg N∙ha^−1^ achieved results similar to, or better than, the non-inoculated control receiving 120 kg N∙ha^−1^, highlighting its potential to reduce synthetic nitrogen fertilizer use while enhancing productivity. This study underscores the important role of nitrogen-fixing bacteria as a sustainable alternative for fertilizing crops like maize.

**Figure 1:**
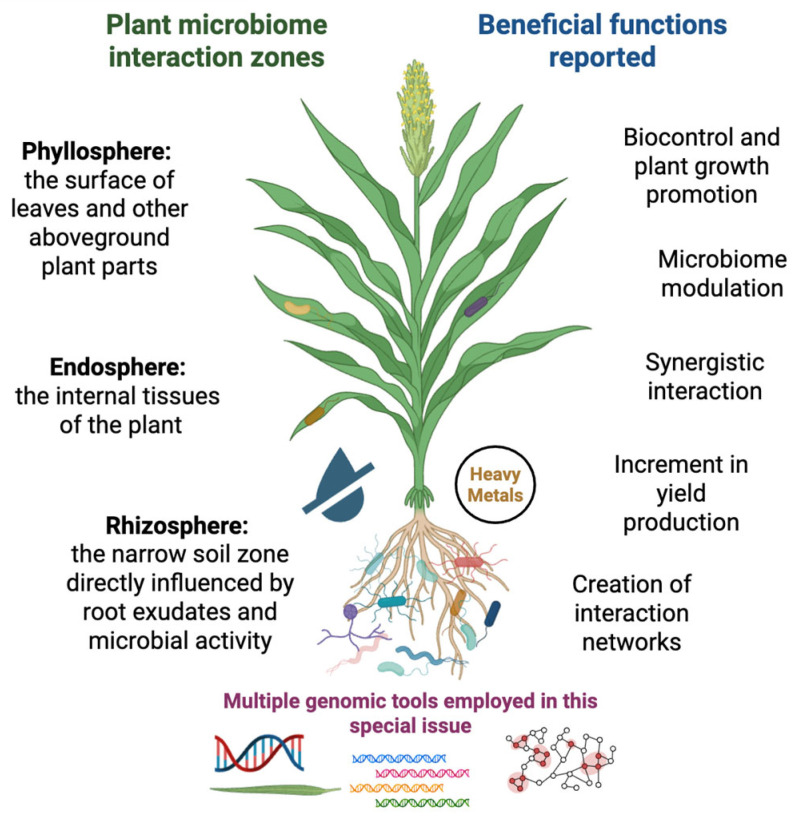
A general overview of plant microbiome interaction zones and selected beneficial functions reported in the 11 studies of this Special Issue.

Figure 1 illustrates the different zones of microorganism–host plant interactions, as well as the beneficial functions detected in the various studies of this Special Issue. In addition, different “omics” tools were reported in these works, including genome sequencing, metagenomic analyses, molecular markers such as ITS and 16S rRNA gene sequences, and other in silico studies of plant microbiome interactions.

## 3. Conclusions

The studies published in this Special Issue further reinforce the concept of the microbiome as a “second genome” associated with the host, encompassing crops such as rice, maize, tomato, cotton, passion fruit, sorghum, and even aquatic plants. This second genome aligns with the hologenome concept, which integrates both host and microbiome genomes. Viewed as a broader ecological entity, this association constitutes the holobiont, in which both partners mutually benefit from their interactions. Such beneficial relationships play a crucial role in enabling plants to cope with diverse environmental and biotic stresses, including heavy metal toxicity, drought, heat, and infections by fungal pathogens such as *Fusarium* and *Verticillium*. Within the microbiome, distinct interaction networks are formed and stimulated by nitrogen-fixing bacteria, *Bacillus* spp., and mycorrhizal fungi, which collectively and synergistically enhance plant nutrition and growth. Overall, the plant microbiome represents a complex and dynamic system, whose multipartite interactions we are only beginning to unravel.
